# Mechanism-Aware Deep Learning for Polar Reaction Prediction

**DOI:** 10.1021/jacs.5c16838

**Published:** 2025-10-22

**Authors:** Ryan J. Miller, Alexander E. Dashuta, Brayden Rudisill, David Van Vranken, Pierre Baldi

**Affiliations:** † Department of Computer Science, 8788 University of California, Irvine, Irvine, California 92697, United States; ‡ Department of Chemistry, University of California, Irvine, Irvine, California 92697, United States

## Abstract

Accurately predicting chemical reactions is essential for driving innovation in synthetic chemistry, with broad applications in medicine, manufacturing, and agriculture. Yet reaction prediction remains a complex problem that is both time-consuming and resource-intensive for chemists to solve. Deep learning offers an appealing solution by enabling high-throughput prediction, but most existing models are trained on the US Patent Office data set and treat reactions as recipes or overall transformations—mapping reactants directly to products with limited mechanistic insight. To address this, we introduce PMechRP (Polar Mechanistic Reaction Predictor), trained on the PMechDB data set of polar elementary steps that capture electron flow and mechanistic detail. To broaden coverage and improve generalization, we augment PMechDB with combinatorially generated reactions and train models spanning transformer, graph, and two-stage Siamese architectures. In addition to reaction prediction models, we also develop ArrowFinder, a new model that directly predicts arrow-pushing mechanisms for a set of reactants and products. Our best-performing approach is a hybrid pipeline that combines an ensemble of Chemformer models with a two-stage Siamese framework, leveraging the accuracy of transformers while filtering away “alchemical” products using the two-step network and generating mechanistic annotations using ArrowFinder. This approach achieves strong predictive accuracy while also providing interpretable predictions. We evaluate performance across multiple benchmarks: PMechDB test splits, a curated USPTO subset from the Open Reaction Database, and a human benchmark of mechanistic pathways from an intermediate-level textbook.

## Graphical abstract

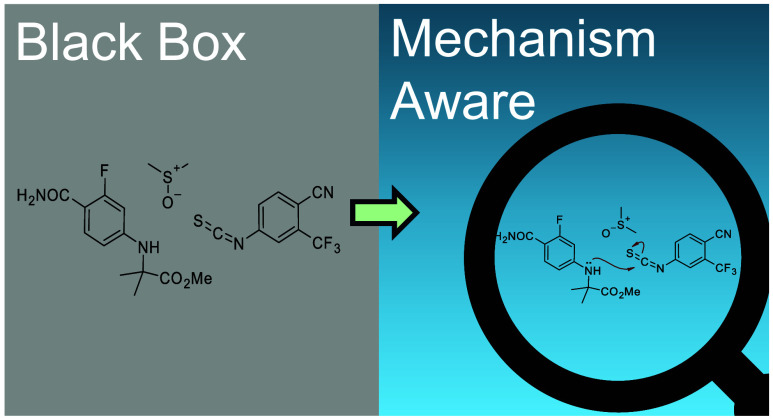

## Introduction

Three main approaches exist for the prediction of chemical reactions: quantum chemistry based methods,^1–4^ rule-based methods,^5^ and machine learning (ML) based methods.^6–17^ Quantum chemistry methods offer highly accurate predictions of chemical properties, but their significant computational cost renders them slow and limits their use for broad, high-throughput reaction prediction. On the other end of the spectrum, rule-based models offer rapid predictions, but suffer from inflexibility. Because chemical reactions span an infinite and extremely complex space, encoding them into a fixed set of rules is inherently limiting. Such systems often fail when they encounter reactions outside their predefined scope. For a balance between precision and speed, ML models offer both flexibility and scalability, making them well-suited for application across larger chemical systems and data sets. Countless ML models have been devised for tasks such as reaction yield prediction,^6^ reaction classification,^7^ chemical property prediction,^8,9^ and both forward and reverse reaction prediction.^10–17^

Although ML models offer high-throughput and highly adaptable chemical prediction, a significant drawback lies in their lack of interpretability in comparison to quantum chemistry based methods. The predominant approach of training models on the USPTO (US Patent Office) data set,^18^ means many ML models predict reactions as overall transformations. This results in a black-box scenario, where predicted products emerge directly from reactants without insight into intermediate transition states. Although these models may achieve high accuracy on the USPTO data set, their outputs pose challenges for organic chemists, who typically reason through chemical synthesis via arrow-pushing mechanisms rather than overall transformations. An example of the overall transformation versus a mechanistic elementary-step approach can be seen in Figure 1. The elementary-step approach breaks the overall transformation down into a sequence of arrow-pushing steps, which illustrate the flow of electrons and the shifting of atoms.

**Figure 1:**
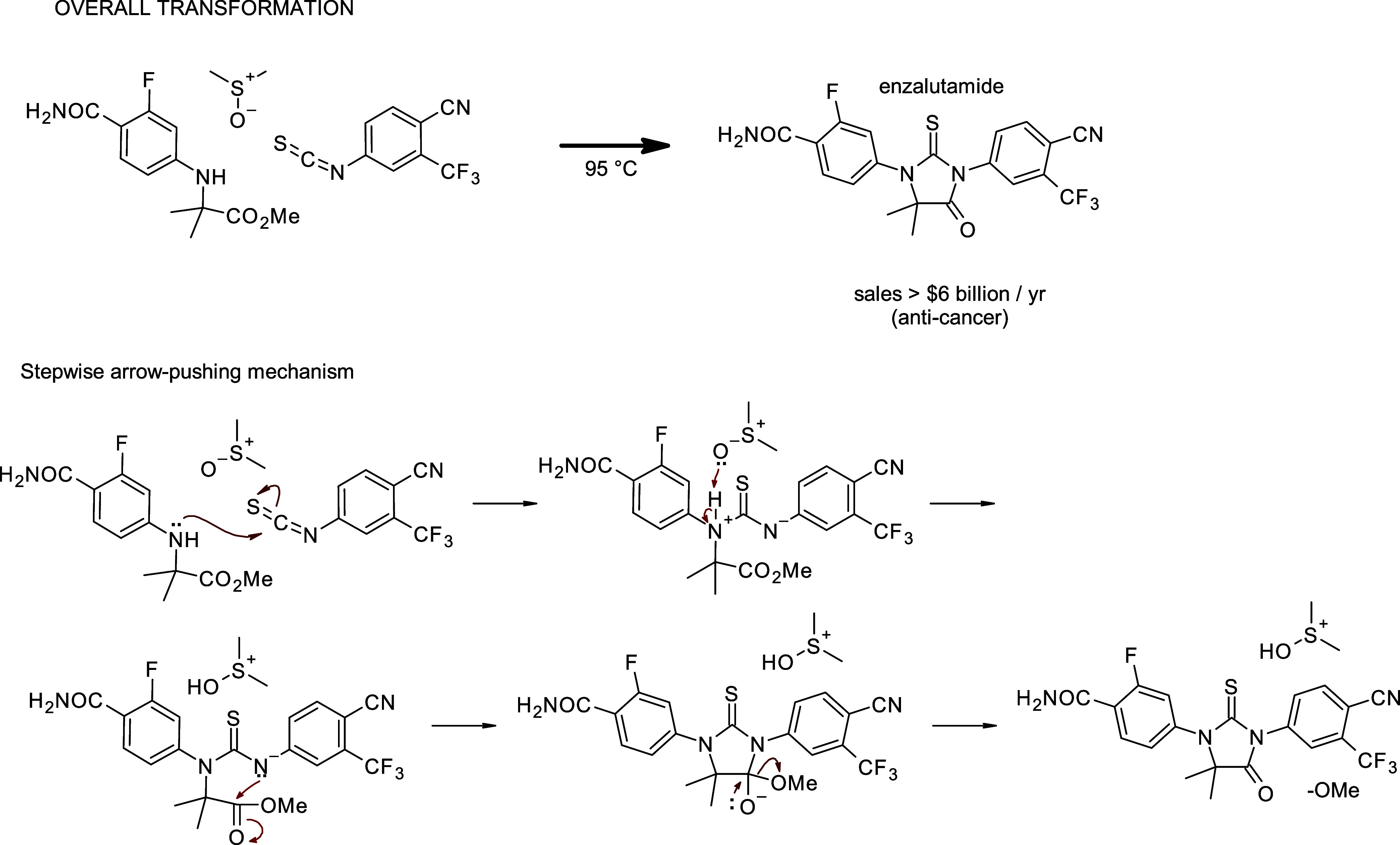
Example of an overall transformation vs an elementary-step approach. This is the final reaction step in the synthesis of enzalutamide, a drug used to treat prostate cancer that generates over $6 billion a year in revenue.^19^

By thinking of reactions as occurring through elementary steps, organic chemists can reason about the underlying driving forces of a reaction. These mechanistic insights help explain phenomena such as unexpected side products or variations in product yield. Figure 2 illustrates the importance of understanding these intermediate steps in a mechanistic pathway where the purity of the final products was affected by a side reaction. When training ML models to forecast elementary-step reactions, we effectively guide them to emulate organic chemists’ thought processes, thereby generating predictions that are more easily interpretable and serve as practical aids for organic synthesis design.

**Figure 2:**
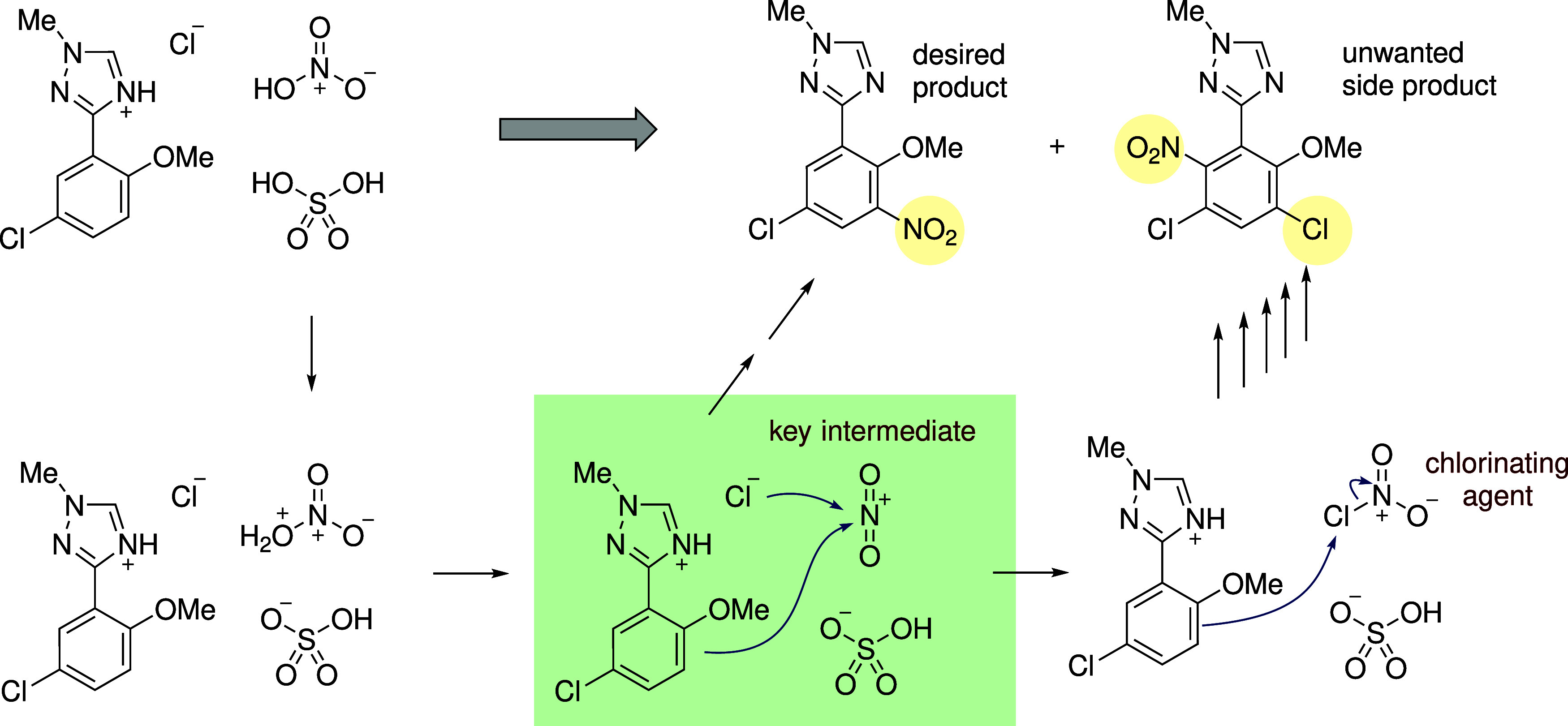
A side reaction occurring at an intermediate step in the synthesis of the autoimmune drug Deucravacitinib, generated unwanted side products due to competing addition of chloride anion to the key NO_2_^+^ intermediate. This led to a decrease in overall purity of the products.^20^

A further limitation of the popular USPTO data set is the presence of a substantial number of unbalanced reactions. Training on such reactions can lead to models that produce unbalanced predictions, which poses particular problems for pathway prediction. When expanding the tree of plausible reactions during a pathway search, it is critical that all atoms are accounted for in each step—otherwise, the predicted pathways may “lose” atoms, creating branches which do not have access to all available reactive atoms. In contrast, data sets like PMechDB, which are both balanced and mechanistically annotated, provide a more chemically rigorous foundation for model training.

## Data

### Manually Curated

To develop predictive models for polar reaction mechanisms, we trained on the PMechDB manually curated data set. This data set consists of approximately 13,000 polar elementary steps, each balanced, with reactive atom maps and arrow-pushing annotations. Each step represents a single elementary-step polar reaction, and is manually verified by a team of organic chemists. These entries have been collected through manual curation from a diverse array of chemistry literature and textbooks.^21^

### Combinatorial Reactions

In addition to the manually curated data, we incorporated a combinatorial data set of 48,761,980 kinetically plausible proton transfer steps, generated by pairing over 7600 acids and 7600 bases.^22^ Each acid and base was assigned reactive atom mappings. When paired together, these mappings can be used to generate arrow-pushing mechanisms between them. For every elementary step generated, the rate constants were estimated from aqueous p*K*_a_ values using the Eigen relationship,^23^ and only reactions with *k* ≥ 10^3^ M^–1^ s^–1^ were retained. Most acids and bases were taken from the structurally diverse DataWarrior data set,^24^ with 98% having p*K*_a_ values in the titratable range (0–14). This data set was used to augment the PMechDB training sets and assess whether adding combinatorial proton transfer steps improves model performance. Additional information about the data set curation is available in the Supporting Information, and the underlying methodologies of the combinatorial generation are explained in greater detail in Vranken et al.^22^

### Training and Testing Splits

To train and evaluate the models, we first constructed a random 80/10/10 train/validation/test split on all the manually curated PMechDB reactions. To better quantify variance in performance estimates, we generated four additional splits. In each case, the validation set was resampled from the training set such that it does not overlap with the validation reactions used in the previous splits. The test set was held fixed across all five curated splits, while the training and validation boundaries were varied. This yielded five distinct data splits used to train and evaluate models. We refer to these as the manually curated data splits. It should be noted that these evaluations reflect estimated performance on reaction classes the model has already encountered, rather than performance on entirely novel reaction types.

For augmentation experiments, we randomly sample 10,000 combinatorially generated reactions from the 48 M proton transfer steps and add them into the training portion of each manually curated data split. We refer to these as the mixed data splits. All models were trained and assessed on both manually curated and mixed splits of the data.

### Human Benchmark Pathway Data Set

To assess the model’s ability to predict full reaction pathways, we curated a data set of 350 mechanistic pathways from an intermediate-level organic chemistry textbook.^25^ The pathway depths range from 1 to 7 steps. Each pathway consists of a set of reactants, a target product, and up to 6 plausible key intermediate structures, such that a pathway of depth d includes d–1 intermediates. To establish a human benchmark, these reactants were assigned to upper-division chemistry students who were asked to predict the structure of the final product based on the provided reactants and target molecular formula. 70 students were assigned 5 pathways each. For 149 of the 350 assigned problems, the student’s answer matched the molecular formula but did not match the correct product structure. Of these 149 incorrect product structures, 40 were inconsistent with any known transformation and did not appear to arise from a mechanistic analysis (could not arise through any arrow-pushing mechanism). For 21 of the 350 assigned problems, the student’s answer did not match the correct molecular formula. To provide a more cautious estimate of student performance, we remove these 61 predictions as they did not appear to involve meaningful student effort. This filtering resulted in 289 problems, of which 180 were answered correctly—yielding an undergraduate (UG) benchmark accuracy of 62.3%. Since omitted submissions may reflect an inability to predict the mechanism, this figure should be viewed as a generous estimate of UG performance. Additional details on the curation and evaluation process are provided in the Supporting Information.

### Open Reaction Database Test Set

To provide a challenging test set to assess the model’s ability to generalize to additional unseen chemical transformations, we randomly selected 20 data sets from the Open Reaction Database (ORD). This random sampling ended up being 19 U.S. patent office data sets, and 1 data set from an optimization study in the Doyle group.^26^ From each of the 20 data sets, we randomly selected 20 transformations, creating a total of 400 steps. We assessed each entry for a complete and correct set of reactant structures, temperatures, and a target product structure. Approximately 60%, or 241 out of 400 transformations were deemed to be unsuitable for mechanistic prediction, some with more than one type of issue. The issues fell into six primary categories: i. mis-drawn product or starting material structure (20%, 79 out of 400), ii. missing reactants (19%, 77 out of 400), iii. multistep sequence (12%, 48 out of 400), iv. incompatible reagent present (12%, 47 out of 400), v. salt structure not depicted (9%, 36 out of 400), and vi. erroneous additional reactant (4%, 14 out of 400). All of the randomly chosen transformations from the Doyle optimization/screen (5%, 20 out of 400) were removed due to low yields (0.20–9.9%). Lastly, reactions which contained multiple target products were removed. The remaining 133 transformations were used for testing the models. Alkali and alkaline earth cations were excluded from the reagent sets, and solvents were added to the reactants side. We refer to this cleaned test set as the ORD test set. More detailed examples of the issues encountered are contained in the Supporting Information.

## Methods

Here we describe several different machine learning approaches for predicting polar elementary-step mechanisms. These methods fall into two distinct categories: the reactive atom two-stage approach, and the single-step seq-to-seq or graph-to-seq prediction methods.

### Single-Step Prediction

We evaluated several transformer-based and graph-based models that treat reaction prediction as either a single-step sequence-to-sequence or graph-to-sequence translation problem, mapping reactant SMILES strings to product SMILES. These include Molecular Transformer,^27^ Chemformer,^28^ T5Chem,^29^ and Graph2SMILES.^30^ While these models have demonstrated strong performance on benchmark data sets like USPTO, they do not provide arrow-pushing information, fail to enforce chemical validity, and in the case of sequence-to-sequence models, lack permutation invariance. The Supporting Information contains additional details regarding the training of each model.

### Two-Stage Prediction

In contrast to black-box single-step models, we implemented a two-stage architecture^10^ that explicitly models electron flow via reactive atom identification and arrow-pushing mechanism enumeration. The model first predicts source (electron-donating) and sink (electron-accepting) atoms using dedicated classifiers trained on atom-level features. These predicted sites are then used to generate possible mechanisms via OrbChain.^10,31,32^ Each mechanism is evaluated by the shared layers of a Siamese network, which assigns a plausibility score used to rank the candidates. This approach provides easily interpretable predictions with mechanistic rationale for each step. Additional details describing this methodology are provided in the Supporting Information.

### Mechanism Reconstruction (ArrowFinder)

We introduce ArrowFinder, a model for predicting arrow-pushing mechanisms. This model adapts the methodology of the two-step Siamese architecture to accept both reactants and products as input, and propose a plausible arrow-pushing mechanism to transform the reactants into the products. The model uses the reactive atom predictors from the two-stage model to propose source and sink atoms, and then it enumerates all possible mechanisms between the source and sink atoms using OrbChain. It keeps track of which mechanisms successfully recover the products, and then applies the Siamese ranker model to assess the plausibility of those arrow-pushing mechanisms, selecting the most likely one. ArrowFinder allows us to take the predictions from an arbitrary, noninterpretable reaction prediction model such as Chemformer, and transform the predictions into arrow-pushing mechanisms. This addresses several of the interpretability drawbacks of the single-step prediction methods.

### Hybrid Approach

Drawing from the strengths of both single-step and two-stage prediction methods, we propose a hybrid approach that integrates the predictive strength of a 5-ensemble of Chemformer models with the mechanistic validity of the two-stage model. While the Chemformer ensemble yields strong predictive performance, it and other transformer-based models are prone to generating “alchemical” products—those with unbalanced charges or atom counts compared to the reactants. To address this, we apply a postprocessing filter that identifies and discards chemically invalid predictions. For each reaction, if any ensemble-generated product violates charge or atom conservation, it is replaced by the top-ranked prediction from the two-stage model. Because the two-stage architecture is grounded in explicit arrow-pushing mechanisms, it ensures greater mechanistic plausibility. As a result, the final hybrid predictions are now sanity checked for “alchemical” products. As a final step, we can optionally apply ArrowFinder to generate arrow-pushing mechanisms for the Chemformer-based predictions in order to annotate them with arrow-pushing mechanisms.

## Results and Discussion

### Performance on Manually Curated Data Set

We train all models on 5 folds of the manually curated data set. The results comparing the performance of the trained models on the test splits can be seen in Table 1.

**Table 1:** Top-N Accuracy of Trained Models (Mean ± Std)

Model Type	Top-1	Top-3	Top-5	Top-10
Best Two-Stage Siamese	57.0 ± 0.008	76.2 ± 0.009	81.2 ± 0.01	84.1 ± 0.007
MolTransformer	50.4 ± 0.008	62.6 ± 0.009	65.5 ± 0.009	65.7 ± 0.009
T5Chem	62.2 ± 0.02	74.2 ± 0.01	77.2 ± 0.01	79.6 ± 0.008
Graph2Smiles	76.6 ± 2.4	83.0 ± 1.6	83.8 ± 1.4	84.3 ± 1.3
Chemformer	80.0 ± 0.01	88.2 ± 0.007	89.1 ± 0.006	89.3 ± 0.005
5-Ensemble Chemformer	81.6 ± 0.002	90.8 ± 0.003	91.5 ± 0.002	91.5 ± 0.002
Hybrid	81.7 ± 0.002	92.1 ± 0.004	93.7 ± 0.003	95.1 ± 0.003

Although the Siamese two-stage model allows for improved interpretability due to its direct prediction of arrows, Chemformer yielded the most accurate predictions among all nonensemble models. Performance of the Chemformer model is significantly improved through ensembling, with further gains achieved by integrating it with the two-stage model in the hybrid approach. The hybrid model demonstrates superior performance, achieving a top-10 accuracy of 95.1%.

### Performance on Mixed Data Set

Furthermore, we assess the performance benefits of the combinatorial reactions by training the same models on 5 folds of the mixed data set. The accuracy results can be seen in Table 2.

**Table 2:** Top-N Accuracy of Trained Models on Mixed Data Set (Mean ± Std)

Model Type	Top-1	Top-3	Top-5	Top-10
Best Two-Stage Siamese	54.8 ± 0.01	75.0 ± 0.007	80.2 ± 0.008	84.5 ± 0.005
MolTransformer	53.5 ± 0.008	65.9 ± 0.008	68.4 ± 0.008	68.6 ± 0.007
T5Chem	64.2 ± 0.01	75.4 ± 0.01	78.2 ± 0.007	80.8 ± 0.006
Graph2Smiles	78.3 ± 0.3	84.0 ± 0.3	84.6 ± 0.4	85.3 ± 0.2
Chemformer	81.2 ± 0.009	89.1 ± 0.007	89.6 ± 0.007	89.7 ± 0.007
5-Ensemble Chemformer	82.2 ± 0.004	91.4 ± 0.003	91.9 ± 0.002	91.9 ± 0.002
Hybrid	82.3 ± 0.004	92.7 ± 0.003	94.1 ± 0.002	95.5 ± 0.003

The addition of combinatorial reactions led to an increase in top-k prediction accuracy across all models except for the two-stage model, which experienced mixed results. The largest improvement was observed for the MolTransformer, which gained nearly 3% in top-10 accuracy, while most other models saw more modest gains. T5Chem, Graph2Smiles, and Chemformer improved top-10 accuracy by 1.2, 1.0, and 0.4% respectively. Although the two-stage Siamese model, experienced a 0.4% gain in top-10 accuracy when trained on the mixed data set, it had significant decreases across the other top-k accuracies with the largest decrease being a 2.2% drop in top-1 accuracy.

Because of the performance decreases on the two-stage model, the hybrid model in Table 2 was constructed in the following way: rather than using both components trained on the same mixed data set, it combines the two-stage model trained exclusively on the manually curated data set (where it performs best on lower top-*k* values) with the 5-Ensemble Chemformer trained on the mixed data set (which benefits across all top-k from the combinatorial augmentation). Evaluated in this configuration, the hybrid model achieved a small additional gain of 0.4% in top-10 accuracy on the mixed data set when compared to the manually curated data set. This version of the hybrid model is selected as our best-performing model and is used later for pathway analysis. A possible explanation for the overall performance gains is that the added combinatorial reactions expand the diversity of possible reactants and products, helping models generalize better and reducing overfitting to the relatively small training set of approximately 10,000 manually curated reactions.

### Performance of ArrowFinder

In addition to evaluating the performance of the reaction prediction models, we evaluate the performance of ArrowFinder on predicting arrow-pushing mechanisms. When we take the true reactants and products from the test set, ArrowFinder was able to generate ground-truth arrows which correctly recover the product in 1331 out of 1337 reactions, or in 99.55% of cases. Analyzing the predicted arrows, the arrows exactly matched the arrow-pushing annotation in 1230 of the test reaction cases. The 101 cases where the model generated arrows which recovered the products, but did not match the exact arrow-pushing annotations were analyzed. It was found that in all cases where the products were recovered, the discrepancies were purely representational: in some cases, the predicted arrows corresponded to the same underlying mechanism but used a slightly different numbering or ordering, while in other cases the arrows described an alternative, but functionally equivalent mechanism.

ArrowFinder was also evaluated on the 5-Ensemble Chemformer model predictions. Among the 1234 predictions made by the ensemble which recovered the true-products, ArrowFinder was able to generate arrows which recover the products in 1,233 cases, leading to a recovery rate of 99.92%. Among all 2858 predictions generated by the ensemble of models which survived atom and charge balance filtering, ArrowFinder was able to generate arrows to recover the products in 1968 cases, or 68.86% of the time. Due to the nature of transformer models, the predicted products do not always have a simple or plausible arrow-pushing mechanism available, and in such cases, it is difficult for ArrowFinder to propose a matching mechanism. However, in cases where the model predicts the correct elementary-step products, ArrowFinder is able to recover the mechanism with a high degree of accuracy.

Using these results, we carried out a preliminary experiment to explore whether ArrowFinder can serve as a plausibility heuristic. Specifically, we take the outputs of the 5-model Chemformer ensemble and pass the predictions to ArrowFinder, which (i) reconstructs arrow-pushing steps when possible and (ii) provides a plausibility signal (“mechanism-recoverable” vs not). We then use this plausibility signal to rerank the Chemformer predictions: predictions with recovered mechanisms are ranked first, followed by those without. Applied to fold0 of the mixed data set, this ArrowFinder heuristic yielded a modest improvement of 1.2% in the top-1 accuracy of the Chemformer ensemble. The full results can be found in Table 3. Although this gain is limited and observed on a single fold, it suggests that mechanism recoverability may serve as a useful filter for identifying more plausible predictions. Further work will be needed to assess the consistency of this effect across data sets and to explore whether incorporating such a filter into pathway search can help prune away implausible pathways.

**Table 3:** Top-N Accuracy of Ensemble Chemformer Using ArrowFinder Reranking

Model Type	Top-1	Top-3	Top-5
5-Ensemble Chemformer	81.8	91.3	91.7
5-Ensemble Chemformer w/ArrowFinder Reranking	83.0	91.6	91.7

### Human Benchmark: Results and Analysis

In addition to predicting single steps, we evaluate the ability of the hybrid model on mechanistic pathways. We took the human benchmark pathway data set of 350 mechanistic pathways (containing reactants, targets, and key intermediate structures) with sizes between 1 and 7 steps and evaluated the performance of the best-performing hybrid model. To predict pathways, the predicted elementary steps were chained together starting from the reactants. We perform a depth-first search using a branching factor of 5 for depths 1–4, and a branching factor of 3 for depths 5–7. The model stops searching down a path once it encounters the target molecular formula or reaches the max depth. After the model enumerates all paths it can find to the target molecular formula, it then picks the path with highest sum of step scores as its final prediction. We consider the target to be recovered if the rank-1 path of the model matches the exact product structure.

To provide another point of comparison, we also ran a pathway search where the paths terminate if the model finds the exact target structure, rather than the target molecular formula. This leads to an increased rate of true targets being recovered, but provides the model with more information than the students had access to (the students were only provided with target molecular formula information). This is not a fair comparison against the students, but provides an “idealized” case where the model is always able to discern the correct target structure from the molecular formula. We present the results of our best-performing hybrid model using both search methods in Table 4. In all tables, the term depth refers to the number of elementary steps in the ground-truth pathway from the benchmark.

**Table 4:** Exact Targets Recovered at Different Depths^a^,^b^,^c^

Depth	Total Pathways	MF Search	ES Search
1	37	30	30
2	113	78	89
3	108	66	89
4	35	17	22
5	38	13	18
6	16	6	7
7	3	1	2
all	350	211	257

a Depth: Pathway length (number of steps) in the benchmark data set.

b MF Search: Number of ground-truth targets recovered by molecular formula search.

c ES Search: Number of ground-truth targets recovered by exact structure search.

While the hybrid model’s 60.3% accuracy is slightly below the 62.3% achieved by students in the UG benchmark, the two performances are quite comparable. When given access to the exact product structure, the model finds pathways leading to the products in 73.4% of cases. This gap suggests that the model has difficulty identifying which pathway should be ranked first: it can generate pathways that recover the exact product structure, but it struggles to discern which pathway is most plausible as the “best” pathway.

We compute additional statistics on the exact structure search pathways. We compute the max number of nodes explored, the average number of solutions found per pathway, the average step rank of predicted pathways, and the percentage of intermediates recovered. These additional pathway stats are provided in Table 5

**Table 5:** Additional Statistics of Exact Structure Search Pathways^a^,^b^,^c^,^d^,^e^

Depth	MXNE	MSF	ASR	%IR
1	6	1.7	1.7	
2	31	2.8	1.6	77.0
3	145	5.9	1.7	79.2
4	708	8.1	2.0	62.9
5	334	3.6	1.4	55.3
6	547	8.4	1.8	53.8
7	1036	16.7	1.6	44.4

a Depth: Pathway length (number of steps) in the benchmark data set.

b MXNE = Max Nodes Explored.

c MSF = Mean Solutions Pathways Found Per Reaction.

d ASR = Average Step Rank of Predicted Pathway.

e %IR = Percentage of Intermediates Recovered.

The model successfully recovers 459 out of 684 annotated key intermediate structures, corresponding to an overall intermediate recovery rate of 67.1%. We note the “best” predicted pathways are generally composed of highly ranked elementary steps: across all depths, the average step rank remains close to 1, with the worst case being 2.0 at depth 4. This indicates that, once a correct step is identified, the model is usually able to place it near the top of its ranking.

To further assess model performance, our team of trained organic chemists manually reviewed each predicted pathway from the exact structure pathway search and evaluated its chemical plausibility. This involved looking through each predicted elementary step, and providing plausibility annotations for all pathways. Steps deemed implausible contain annotations explaining the reasoning behind the classification. A summary of the results can be seen in Table 6.

**Table 6:** Plausibility of Proposed Pathways during Exact Structure Search

depth	total pathways	# found exact structure	Plausible pathways	% Plausible
1	37	30	30	81
2	113	89	82	73
3	108	89	58	54
4	35	22	16	46
5	38	18	8	21
6	16	7	2	13
7	3	2	0	0

We observed that overall plausibility decreased with increasing pathway length-from 81% for 1-step pathways to 0% for the 7-step pathways. The result is expected, as at higher depths it becomes increasingly difficult to continually predict plausible elementary steps. Additionally, deeper pathway searches tend to yield many alternative solutions, making choosing the “best” pathway more difficult for the model. In most cases, implausible pathways were associated with one (or more) implausible steps as opposed to an improper order of steps. Some of the errors fell into common categories: improper or missed proton transfers (19/154), improper SN2 reactions at carbon (11/154), improper use of bond to the attacking atom instead of a lone pair on the attacking atom (8/154), errors in generation of electrophiles for electrophilic aromatic substitution (6/154), and concerted substitution reactions at, for example, lithium and phosphorus (5/154). The errors associated with electrophilic aromatic substitution probably arise from the training data, as is common practice for mechanistic depictions, spectators (e.g., benzene, toluene, etc.) are often excluded from depictions of arrow-pushing steps. These five categories correspond to panels (A)–(E) in Figure 3.

**Figure 3:**
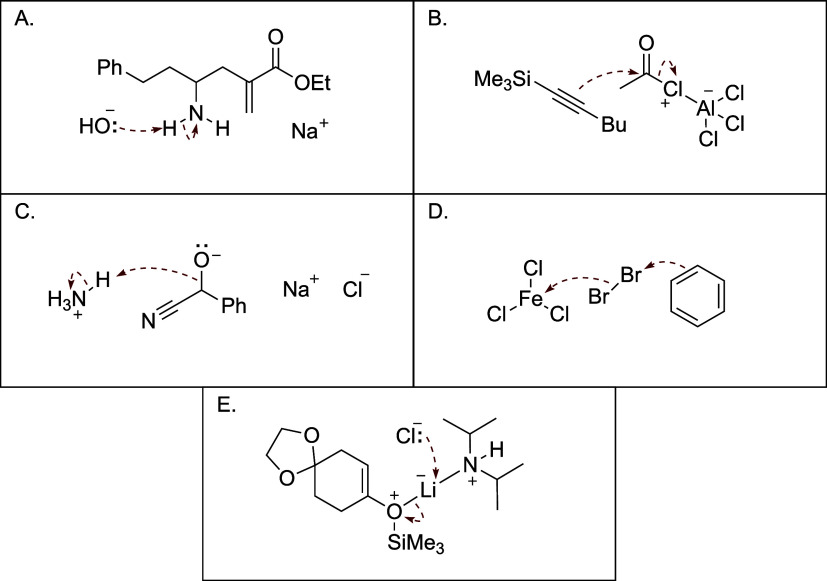
Examples of implausible mechanistic steps: (A) Improper proton transfer; (B) improper S_N_2 displacement on carbon; (C) improper use of bond instead of a lone pair; (D) improper generation of electrophilic for electrophilic aromatic substitution; and (E) concerted displacement reactions at lithium and other metals.

Notably, the system generated plausible multistep pathways for several interesting transformations, including a six-step enol ether hydrolysis, a five-step carbodiimide coupling, a five-step DMAP-catalyzed acyl substitution, and a four-step acid-catalyzed Peterson olefination (Figure 4).

**Figure 4:**
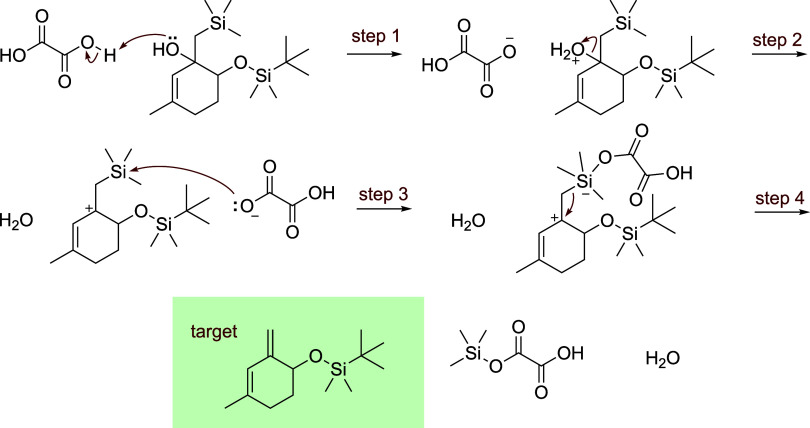
Example of correctly predicted Peterson olefination.

For additional analysis, we make the predicted pathways, individual pathway statistics, and plausibility/implausibility annotations free for download.

### ORD Test Set: Results and Analysis

To evaluate the generalization capabilities of the hybrid model on a more challenging benchmark, we tested it on a set of USPTO reactions extracted from the Open Reaction Database (ORD). The reactions were curated to ensure that each entry contained a complete set of reactant structures and a single target structure. Importantly, this data set includes transformations and molecules not covered by PMechDB, presenting a significant challenge for the model. We performed a beam search of depth 5 with a branching factor of 5 across 133 reactions, terminating the search once a pathway to the target was identified. The system successfully recovered products for 35 of the 133 reactions (26.3%).

Targets were not recovered for particularly complex cases, such as palladium-catalyzed Stille couplings, nonstandard reagent representations (e.g., LiAlH_4_ represented as Al^4+^ + 4H^–^), or one-electron processes such as benzylic bromination. Among the recovered pathways, 21 of 35 (60%) were determined to be chemically plausible. Plausible recoveries included transformations such as base-promoted SN2 reactions and acylations.

Although this benchmark highlights the difficulty of generalizing to a new data set, the results remain promising. Despite containing mechanisms and molecules absent from the PMechDB data set, the hybrid model was able to identify viable pathways for over a quarter of the targets, and more than half of these solutions were found to be chemically plausible.

### Alternative Mechanistic Data Set Results

Recent work by Coley et al.^16,17^ and Jung et al.^33^ has algorithmically converted USPTO reactions into mechanistic steps using templates. To compare our methods to existing state-of-the-art mechanistic models, we train and evaluate Chemformer on the FlowER data sets. In our experiments, it appears Chemformer slightly outperforms the newly introduced FlowER-large model.^17^ On the FlowER test set, Chemformer achieves a top-5 accuracy of 99.38% compared to 99.13% top-5 accuracy for FlowER-large. While FlowER provides important interpretability benefits by predicting the flow of electrons, Chemformer appears to offer slightly higher product prediction accuracy. A more detailed analysis is provided in the Supporting Information.

### PMechRP Web Interface

We provide users with tools to visualize reaction arrow-pushing mechanisms through the SmilesToDepict interface (https://deeprxn.ics.uci.edu/smitodepict/). The Hybrid, Ensemble Chemformer, Two-Stage, and ArrowFinder models are also publicly accessible via an interactive web interface at https://deeprxn.ics.uci.edu/pmechrp. The reaction prediction interface supports two modes: single-step prediction and pathway prediction. For single-step prediction, users input a set of reactants and model parameters, and the system returns the top-N predicted elementary-step mechanisms. For pathway prediction, users provide a set of reactants and a target molecule, and the system conducts a beam search to identify a multistep mechanism connecting the reactants to the target. Search parameters such as branching factor and depth can be adjusted directly through the interface. Lastly, all train/validation/test data sets, pathway data sets, and model checkpoints are available for download at https://deeprxn.ics.uci.edu/pmechdb/download.

## Limitations

We note there are several limitations with the current state of the PMechRP polar reaction system. First, the manually curated PMechDB data set only includes around 13,000 steps. This means the data set is relatively small for training large architectures, and it may be difficult for these models to generalize well to all forms of experimental chemistry. To improve coverage, we augment the data set with combinatorially generated reactions. However, these additional reactions are constructed from a limited set of acids and bases, and while helpful, they do not capture the diversity of chemical space. As such, the overall data set remains limited in scope compared to the complexity of real-world chemistry. Second, the transformer-based models directly translate from reactants to products, without generating the arrow-pushing mechanisms. Although ArrowFinder can be used to propose arrows on transformer predictions in order to provide mechanistic interpretability, it is not guaranteed to find a valid reaction mechanism mapping from reactants to products for all model predictions. Improving source/sink predictors and mechanism enumeration coverage is an important direction for future work to increase mechanism recovery rate. Lastly, by performing hybrid or ensembling methods, or by postprocessing predictions with ArrowFinder or checking for charge and atom balance, the best-performing models have increased computational overhead, and the inference time is comparatively slow.

## Conclusion

We developed and compared several reaction prediction systems for polar mechanisms, demonstrated performance gains from augmenting training data with combinatorially generated reactions, introduced ArrowFinder for arrow-pushing mechanism generation, and curated two new data sets for benchmarking elementary-step prediction. To address the limitations of PMechDB, we are actively expanding coverage to build a master data set that integrates polar, radical, pericyclic, and combinatorial reactions, with plans to release future models trained on these broader data sets. Based on our analysis of polar steps, we developed PMechRP—a system designed to predict polar reactions at the mechanistic level. Our hybrid approach, which combines Chemformer and two-stage architectures, achieves 95.5% top-10 accuracy on the PMechDB test set and a 73.4% target recovery rate on the human benchmark pathway data set. Furthermore, we show that correct Chemformer predictions can be annotated with arrow-pushing mechanisms with high fidelity, demonstrating ArrowFinder’s ability to enhance the mechanistic interpretability of transformer and other general-purpose models. Collectively, these contributions advance the development of interpretable, mechanism-aware reaction prediction systems.

## Supplementary Material



## Data Availability

We make the Two-Stage and ArrowFinder codes available at https://github.com/rjmille3/ArrowFinder. We make all pre-existing models used in the paper freely available at https://github.com/rjmille3/pmechrp_models. The scripts used for combinatorially generating proton transfer steps are available at https://github.com/rjmille3/combinatorial-proton-transfer.

## Notes

Detailed description of the curation processes for all data sets, along with model training parameters and additional experimental results (PDF)
