# Supplementary material for: Mechanism-Aware Deep Learning for Polar Reaction Prediction
Source: J Am Chem Soc. 2025 Oct 22;147(44):41168–76. doi: 10.1021/jacs.5c16838 (PMC12593370; doi:10.1021/jacs.5c16838)
Supplement: Supplementary file 1 [file ja5c16838_si_001.pdf]

Supporting Information
Mechanism-Aware Deep Learning for Polar Reaction Prediction

Ryan J. Miller,[†] Alexander E. Dashuta,[‡] Brayden Rudisill,[†] David Van Vranken,[*,‡] and Pierre Baldi[*,†]

†*Department of Computer Science, University of California, Irvine, Irvine, California 92697, United States*

‡*Department of Chemistry, University of California, Irvine, Irvine, California 92697, United States*

E-mail: david.vv@uci.edu; pfbaldi@uci.edu

Combinatorial Reactions

The combinatorial dataset consists of 48,761,980 kinetically plausible proton transfer steps,[1] generated combinatorially from over 7,600 acids and 7,600 bases. To construct this dataset, we identify source and sink atoms on acids and bases and generate reactive atom mappings for the molecules. We then pair the acids and bases together to generate arrow pushing mechanisms from the reactive atom mappings. For each proton transfer elementary step, rate constants were estimated from aqueous pK_as based on the Eigen relationship[2] and conservative boundaries were chosen for inclusion in the dataset. The majority of the acids and conjugate bases were taken from the DataWarrior dataset.[3] They were structurally diverse with proton donor/acceptor atoms; 98% had pK_a values in the readily titratable range 0-14. Combinatorial proton transfers were also created using about 100 highly acidic and highly basic heteroatom species from the well-known Reich compilation and Guthrie's[4,5] pK_a estimates for protonated carbonyls and tetrahedral intermediates. Proton transfers between heteroatoms with estimated rate constants $\geq 10^3$ M^{-1} s^{-1} — a conservative boundary — were included in the dataset. About 15,000 combinatorial proton transfers were generated from carbon acids and heteroatom bases with rate constants estimated using the Eigen-Bernasconi equation.[6] Steps for proton transfers from carbon with estimated rate constants $\geq 10^{-1}$ M^{-1} s^{-1} were added to the dataset. For generating the mixed datasplits, we sampled 10k reactions from the 48M plausible proton transfer steps, and added them to each of the 5 folds of the manually curated dataset. The pipeline for generating the proton transfer steps can be seen in Figure 1.

In more recent work, we have adjusted several of the acid and base sets, as well as rate constant cutoffs for reaction generation. The new methodology is described in detail in Van Vranken *et al*,[1] and we recommending using this updated datset for Future work. On the deeprxn downlaod page, the "OLD PMechRP Proton Transfer Dataset" refers to the proton transfer reactions specifically used in this manuscript, while "Proton Transfer Dataset" refers to the updated data.

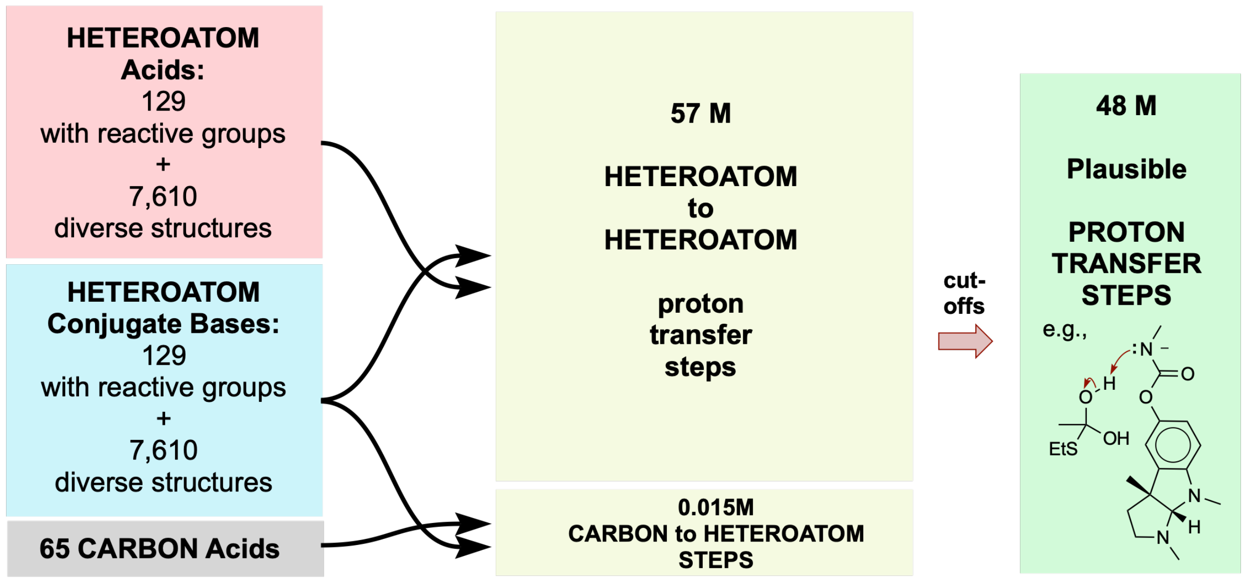

Figure 1: Pipeline for combinatorial reaction generation.

Human Benchmark Pathway Dataset Curation

We chose a well-known intermediate-level organic chemistry textbook *Organic Chemistry, 2nd Ed.*, by Clayden, Greeves, and Warren as a source of pedagogically diverse organic transformations. It contains a number of modern transformations missing from introductory textbooks: allylsilane addition, enol silyl ethers, boron aldol reactions, organosulfur chemistry, organophosphorus chemistry, allylsilane reactions, electron transfer reductions, and heterocycle synthesis. Many of the key transformations include arrow-pushing mechanisms for the key steps.

We translated 1,187 one-step transformations in the Clayden textbook into entries that included reactant(s), temperature, and product combinations for use in testing product prediction. Many of the entries in the textbook had implied secondary workup steps to generate neutral products. Secondary workups were excluded to ensure that the entries were one-step transformations. All entries include scholarly literature references for the transformation. Many of the entries in the textbook were depicted with generic R substituents, so specific examples were selected from the research literature that best matched the transformation. Each entry has an estimate of the minimum number of elementary mechanistic steps needed

to arrive at the product. We refer to this test set as the 1K Test Set.

A subset of the 1K Test Set was used to evaluate students from an upper division organic chemistry class at UC Irvine (Chem 125) at the end of the spring quarter of 2023. The Clayden textbook was recommended, but not required, and the course did not follow the structure of the book. We removed from consideration about 400 transformations that require more than one equivalent of a reactant, either due to stoichiometry or need for a mechanistic acid/base, which yielded 800 entries. We then further reduced this student test set to transformations involving seven mechanistic steps or less, leading to 696 entries. Of these, 60 involved chemistry outside of the scope of the class, so they were not considered for the assignment. The total pool of assignable problems was 636 transformations. Each problem consisted of reactant(s), temperature, and a product molecular formula. The justification for including the molecular formula is that such information is readily available from mass spectrometry and investigators are simply trying to match a product mass to a plausible structure. We refer to this subset of the 1K Test Set as the UG Test Set.

The 70 students in the class were each assigned five different randomly chosen transformations from the UG Test Set. The students were asked to propose a product structure in SMILES format consistent with the reactants, temperature, and the molecular formula of the product. Students were given 3 weeks to complete the assignment. For the purposes of grading, students were told that any structure matching the product formula would receive credit.

For 180 of the 350 assigned problems, about half, the student's product structure was correct. For 21 of the 350 assigned problems, the student's answer did not match the correct molecular formula. For 149 of the 350 assigned problems, the student's answer matched the molecular formula but did not match the correct product structure (Figure 2). Of these 149 incorrect product structures, 40 were inconsistent with any known transformation and did not appear to arise from a mechanistic analysis. Therefore, 109 of 170 incorrect answers appeared to involve student effort. Of the 289 (180 plus 109) problems attempted, 62% of

the products were correctly identified by students. We refer to this performance as the UG Benchmark.

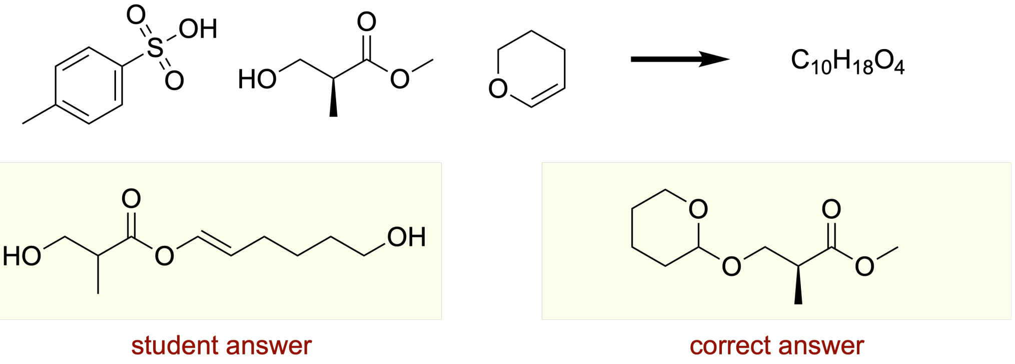

Figure 2: Example of the Transformations Assigned to Students for Product Prediction.

Open Reaction Database Pathway Dataset Curation

We started by randomly selecting 20 datasets (19 from U.S. patent datasets, 1 from an optimization study in the Doyle group). From each of the 20 datasets, we randomly selected 20 transformations from each of the 20 datasets to create a total of 400 transformations. We assessed each entry for a complete and correct set of reactant structures, temperatures, and a target product structure. 60% of transformations were deemed to be INCORRECT, some with more than one type of error (241 out of 400). There were 6 main categories of errors:

i. Mis-drawn product or starting material structure (20%, 79 out of 400)

Example: The name of either the product or structure was incorrect in US Patent 05015740. The structures in ORD match the patent.

Dataset ID: `ord_dataset-d98d003e9abb4b579746c5a361466e14`

Reaction ID: `ord-3a5f189860fc42eeac3bdff0a772886b`

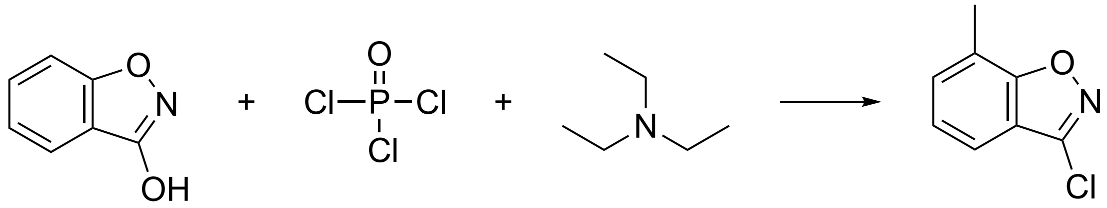

ii. **Missing reagents (19%, 77 out of 400)**

Example: The entry is missing the key reagents: sodium tris(acetoxy)borohydride and acetic acid in 1,2-dichloro-ethane at 20 °C.

Dataset ID: `ord_dataset-c8a367b56b4f406b878f51867b157d19`

Reaction ID: `ord-58d20c52c9e148a6ac57377ccd5a12eb`

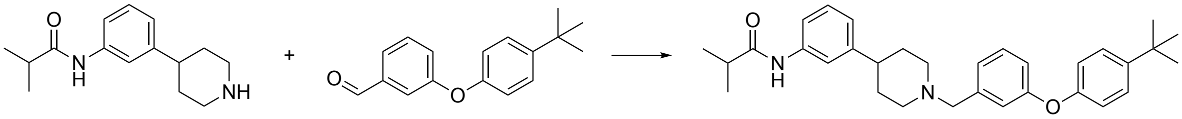

iii. **Multi-step sequence (12%, 48 out of 400)**

Example: The products and/or reactants from each of the four sequential mixing steps are not shown.

Dataset ID: `ord_dataset-18e9ed24dbd44e98b33bdc22aa7580a8`

Reaction ID: `ord-c17bac9bc2394de596d47eb8aefdcd61`

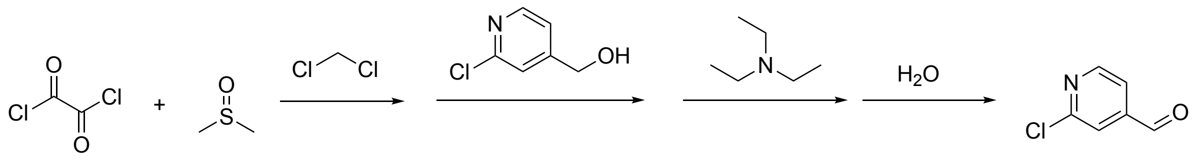

iv. **Incompatible reagent present if the beginning and end of reaction steps isn't specified (12%, 47 out of 400)**

Example: During acetylation of an amine in dichloromethane it isn't clear if water is necessary for the reaction with acetyl chloride.

Dataset ID: `ord_dataset-37b0416f244344a08cf357e851eedf2a`

Reaction ID: `ord-255d83b5206945b1848c756d5bbd653b`

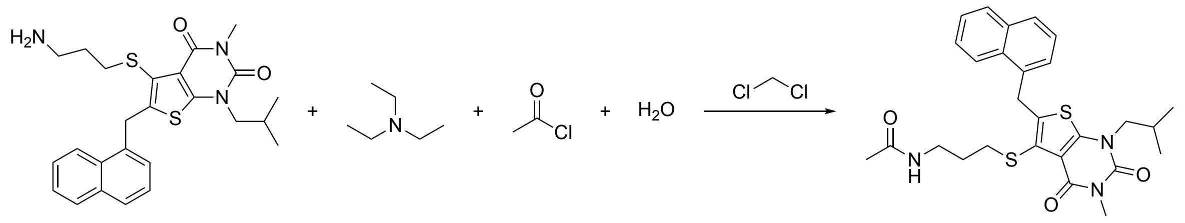

v. **Salt structure not depicted (9%, 36 out of 400)**

Example: The product is not represented as an ammonium chloride salt (with site of protonation).

Dataset ID: `ord_dataset-37b0416f244344a08cf357e851eedf2a`

Reaction ID: `ord-0ff01e3bc5a44865a19a64bdec058a4d`

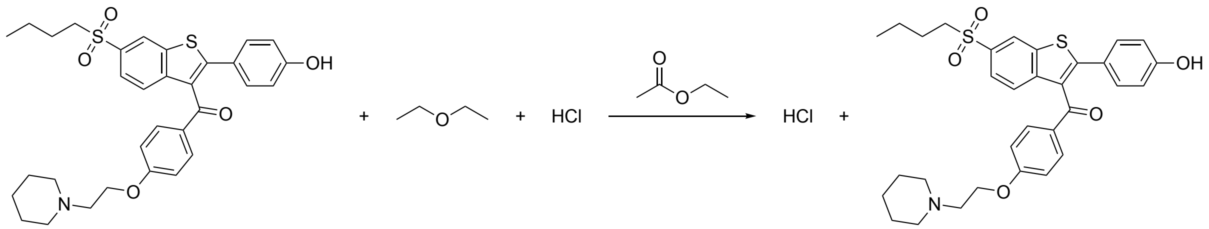

vi. Erroneous additional reactant (4%, 14 out of 400)

Example: The acetate ions do not appear in the product set and the product set contains an additional yttrium atom.

Dataset ID: `ord_dataset-d98d003e9abb4b579746c5a361466e14`

Reaction ID: `ord-67abc74435724ac99d1d6b0e1fad7245`

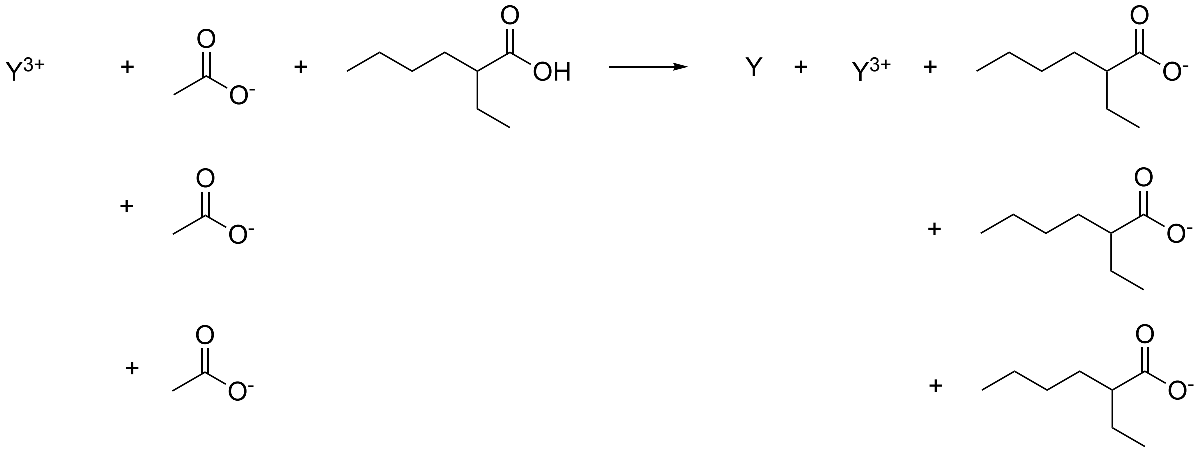

After removing incorrect transformations, all remaining transformations from the Doyle group optimization study dataset[7] were removed due to low yield. Additionally, 7 transformations containing multiple final products were removed to keep data format consistent. For the remaining reactions, the solvents were added to the reactants side of the reaction, and Alkali and Alkaline earth cations were removed. This left a final set of 133 cleaned reactions containing all necessary reactants and solvents, leading to a single final product major structure.

PMechDB Dataset

Here we provide some Figures 3, 4 displaying the the number of atoms and atom types found in the PMechDB dataset[8]

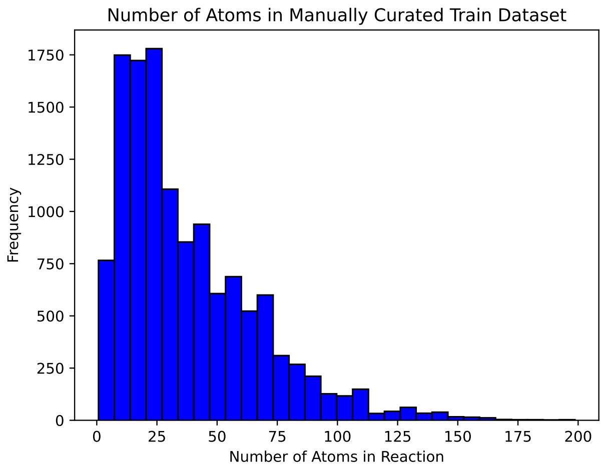

Figure 3: The distribution of the total number of atoms contained in each reaction for the manually curated training dataset.

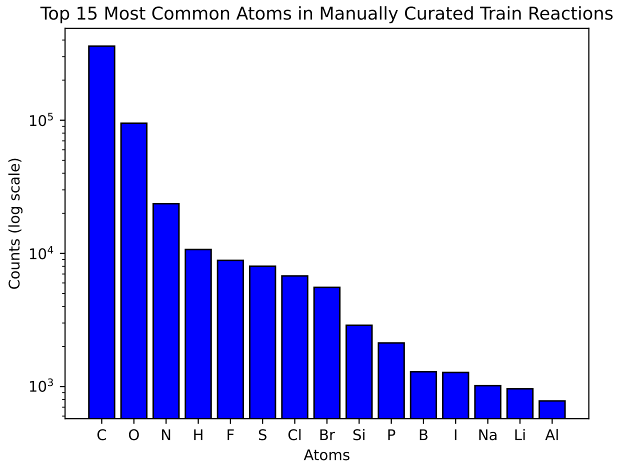

Figure 4: The distribution of atoms for the reactions in the manually curated training dataset.

FlowER Datasets

To benchmark against state-of-the-art methods, we trained and evaluated a single Chemformer model on the mechanistic FlowER dataset.[9]An updated version of the dataset has been curated, but we train and evaluate on the older version which was used in the FlowER paper to make a direct comparison. The Chemformer model was initialized from the USPTO_mixed pretrained checkpoint, and used default hyperparameters with an augmentation probability of 0.1. The model required a vocabulary update for compatibility with the FlowER dataset. To avoid data leakage, we removed 281 FlowER test reactions overlapping with products in the USPTO_mixed training and validation sets, yielding a final test set of 161,721 reactions (originally 162,002). We evaluated Chemformer with 10 beams to obtain a quick lower-bound estimate. For comparison, we use 25 beams to compute the top-k accuracies for Chemformer in the main text. We compare the performance of the two models on the test set in Table 1.

Table 1: Top-K Step Accuracy of Trained Models on FlowER Test dataset

**Model Type**	**Top-1**	**Top-3**	**Top-5**	**Top-10**
Chemformer	89.93	98.93	99.38	99.47
FlowER-large	89.74	98.66	99.13	99.25

The Chemformer model without ensembling, outperforms FlowER accross all top-k accuracies. FlowER still offers significant benefits in that it explicitly ensures the conservation of atoms and charge during model inference, and offers greater mechanistic interpretability. However, out preliminary results indicate the Chemformer model provides more accurate product prediction capabilities.

Scaffold Splits

A scaffold split was performed on the manually curated data. We used the rdkit implementation of murcko scaffolds to generate validation and test sets with unseen molecular scaffolds. We test the generalization abilities of the best performing Hybrid model on the Scaffold Split.

The results can be seen in Table 2

Table 2: Top-N Accuracy of Trained Models on Scaffold Split

**Model Type**	**Top-1**	**Top-3**	**Top-5**	**Top-10**
Hybrid	57.6	70.3	74.3	78.2

The model appears to have reasonable generalization performance on a more challenging test split, achieving a 78.2% top-10 accuracy. The scaffold data splits are available to download at https://deeprxn.ics.uci.edu/pmechrp/download under the name "PMechRP Datasets".

Chemformer Model Filtering Analysis

We evaluated the presence of atom and charge alchemy for a single chemformer model. We use the Chemformer model trained on fold0 of the mixed dataset, and predict with 25 beams. In 1,337 total test reactions, the model generates 33,425 predictions before deduplication. 1,039 of these predictions are invalid SMILES. After deduplication and removing invalid smiles (those which cannot be parsed by rdkit), there was a set of 6,192 unique predictions. Among these unique predictions, 33.6% passed both atom alchemy and charge alchemy filters, while 65.5% violated conservation of atoms, and 25.7% violated conservation of charge. For 7 out of 1,337 test reactions, no predictions remained after charge and atom filtering. A summary of the filtering Results can be found in Table 3

Table 3: Atom and Charge Filtering Results for Chemformer Model

**Total Preds**	**Unique Preds**	**Both Balanced**	**Atom Error**	**Charge Error**
33,425	6,192	2,079	4,036	1,591

Additionally, we noticed that when filtering for atom alchemy, if hydrogens were not explicitly added to both the reactant and prediction molecules, there were some instances where the filter would incorrectly flag predictions with implicit hydrogens as atomically imbalanced. This led to a 0.5% decrease in top-5 accuracy for the 5-ensemble chemformer

model. Note: the top-k results in the main text use the filters with explicit hydrogens added, however, the pathway searches were performed using the older filtering method. This leads to a slight underestimation of the performance of the Hybrid model on the pathway datasets.

Hybrid Model Methodology

To generate Ensemble Chemformer predictions, we combine outputs from five independently trained Chemformer models. For each candidate product, we compute a score by summing the likelihoods assigned by all five models. Since each likelihood lies within $[0, 1]$, a product predicted with full confidence by all models receives a maximum score of 5. We then filter out predictions with charge or atom imbalances and rank the remaining candidates by score to define the Ensemble Chemformer top-k. After filtering, this set often contains fewer than k predictions. In such cases, we supplement the list with non-redundant predictions from the Two-Stage architecture.

During pathway search, each step requires an associated score to calculate the overall pathway score. However, Chemformer and Two-Stage predictions scores are not directly comparable. To bias the system toward the more accurate Chemformer outputs, we rescale Two-Stage scores using a sigmoid mapping into the interval $[0, 0.5]$. This ensures that a single Chemformer model's confident prediction outweighs any Two-Stage prediction, while still allowing the Two-Stage model to contribute when Chemformer confidence is low or inconsistent across models.

Model Training

In this section, we describe the training configurations for the various models. For additional information, we make all source codes used to train the models freely available at `https://github.com/rjmille3/pmechrp_models`. We make the codes for ArrowFinder and the Two-Stage model available at `https://github.com/rjmille3/ArrowFinder`.

Molecular Transformer

We utilized the text-based reaction predictor, Molecular Transformer,[10] which employs a bidirectional encoder and autoregressive decoder coupled with a fully connected network to generate probability distributions over potential tokens. We did not observe performance benefits from pretraining, so the base model was selected and then fine-tuned on the PMechDB dataset. The final 3 model checkpoints were averaged to create the checkpoint used for prediction.

Chemformer

In addition to the molecular transformer, we adopted the Chemformer model,[11] which is another transformer-based reaction predictor. The Chemformer model also employs a bidirectional encoder and autoregressive decoder with a fully connected network to generate probability distributions over potential tokens. The Chemformer model was pre-trained on molecular reconstruction and classification tasks using a dataset of 100M SMILES strings from the ZINC-15[12] dataset. Afterwards, the model was fine-tuned onto various downstream tasks, including forward prediction and retrosynthesis. The pre-training substantially improved the model's generalizability and convergence times on downstream tasks, such as USPTO forward prediction, compared to randomly initialized models. We chose to start from the model fine-tuned on USPTO-mixed since reactants and reagents are not separated in the PMechDB dataset. This model was then fine-tuned on the PMechDB dataset for mechanistic-level predictions. The vocabulary of the model was expanded by 66 tokens to account for unseen atoms in the PMechDB dataset. The default hyperparameters were used besides augmentation probability, which was set to 0.1 for the best performing Chemformer model.

To improve the performance of the chemformer model, we train 5 chemformer models on each data split to create an ensemble. In the benchmark in the main text, the single Chemformer models used 25 beams and saved the top-10 unique predictions. For each ensemble model, we predict with 5 beams per model to keep the total number of beams equal

to the single model and provide a more fair comparison. In order to add some variance to the models, we vary the augmentation probability. For the remaining experiments in this section, we focus on predictions from fold0 of the manually curated dataset. The results of changing the augmentation probability on Chemformer using a beam size of 5 can be seen in table 4

Table 4: Effects of Augmentation Probability on Top-N Accuracy for Chemformer Models

Augmentation Probability	**Top-1**	**Top-3**	**Top-5**
0.0	76.6	84.4	84.5
0.1	**80.1**	**86.0**	**86.2**
0.3	79.5	85.8	85.9
0.5	79.4	84.2	84.2
0.7	78.8	83.6	83.7

We consider the best performing model to be the model with augmentation proability of 0.1, as it achieves the highest accuracy across all top-k. This is the augmentation probability we use when benchmarking single Chemformer models in the main text. To aggregate predictions of the ensemble, we sum the likelihoods from all 5 models for each product. The predictions are then sorted by highest likelihood sum. Ensembling allows the Chemformer models to predict a greater diversity of products, offering increases of around 1% to top-1 accuracy, and an increase of over 2% to the top-10 accuracy. For different ensemble sizes, we include the top-n performing models where we sort the models by their top-5 accuracies. The impact of varying ensemble sizes on performance for the manually curated dataset is summarized in Table 5.

Table 5: Effects of Ensemble Size on Top-N Accuracy for Chemformer Models

$\boldsymbol{ensemblesize}$	**Top-1**	**Top-3**	**Top-5**	**Top-10**
2	80.6	88.3	88.6	88.7
3	80.6	89.9	90.5	90.7
4	80.9	90.3	91.0	91.0
5	**81.3**	**90.8**	**91.5**	**91.6**

Pretraining the Chemformer models made a large difference in performance, the effects of pretraining can be seen in Table 6.

Table 6: Effects of Pretraining on Top-N Accuracy of Chemformer Models

Model Type	Top-1	Top-3	Top-5
no-pretraining	43.3	56.5	57.2
pretrained on zinc and USPTO Mixed	80.1	86.0	86.2

The large increase in performance from the pretraining, indicates overlap between the USPTO dataset and the PMechDB dataset. This is in stark contrast to radical mechanisms, which exhibited lower performance when using a pretrained model.[13] This suggests that radical reactions are underrepresented in USPTO datasets compared to polar reactions, and that pre-trained transformer models would be expected to have higher performance on polar reactions.

Lastly, we assess the statistical significance of adding combinatorial reactions to the training data. Because we have only five cross-validation folds, we refrain from broad claims about statistical significance across all model families. For the Chemformer 5-ensemble, however, the combinatorial augmentation yields improvements that are statistically significant by a paired, two-tailed t-test on top-k accuracies across folds. The resulting p-values are reported in Table 7.

Table 7: P-values of Two-Tailed T-Test Comparing Top-K Accuracy Differences Between Mixed and Manually Curated Only

Model Type	Top-1	Top-3	Top-5	Top-10
5-Ensemble Chemformer	0.0256	0.0217	0.0496	0.0496

Since $p < 0.05$ for all top-k (including those not shown from top-1 to top-10), we argue that in the case of the Ensemble Chemformer model, the difference between the Mixed and Manually Curated dataset is statistically significant.

T5Chem

Due to the highly related nature of many chemistry prediction tasks, multitask learning can be used to develop robust models which may demonstrate improved learning efficiency and prediction accuracy. T5Chem is one such model, which leverages multitask learning on a transformer architecture to perform 5 different tasks. The T5Chem multi-task transformer architecture is able to perform forward/backwards prediction, reaction yield prediction, reaction classification, and reagents prediction.[14] This architecture was first pretrained with a BERT-like MLM objective on 97 million PubChem molecules. Then, the model was further fine-tuned on 5 different tasks using the USPTO_500_MT dataset. We selected this pretrained model, and fine-tuned it on the manually curated and mixed datasets. We trained for 100 epochs, and used the product task type with default hyperparameters.

Graph2SMILES

Lastly, we employed the Graph2SMILES model[15] for reaction prediction, which replaces the traditional sequence-based transformer encoder with a graph encoder to process molecular graphs as inputs. The model uses a Directed Message Passing Neural Network (D-MPNN) to capture local chemical context, followed by a global attention encoder with graph-aware positional embeddings to incorporate topological information and ensure permutation invariance to SMILES formatting, thus eliminating the need for data augmentation. A transformer-based autoregressive decoder then generates the predictions. We select the GAT model which was pretrained on the USPTO_STEREO dataset. This model was then fine-tuned on the manually curated and mixed datasets. The default hyperparamters were used for training. The vocabulary from the pretrained checkpoint was unable to cover all the reactions of the PMechDB dataset. As such, we created a new vocabulary and had to manually adjust the size and weights of several decoder and output layers to accomodate the new vocabulary size.

Two-Stage Prediction

The two-stage prediction model comprises distinct phases. Initially, the model undertakes the task of predicting reactive atoms within the given reaction. Subsequently, these identified reactive sites are paired to formulate potential reaction mechanisms using OrbChain,[13,16,17] followed by the application of a ranker model to rank the plausibility of these proposed mechanisms. This architectural design yields highly interpretable predictions, enabling a granular understanding of the model's rationale. When generating predictions, users can discern precisely which atoms are deemed reactive, and they can view the precise arrow-pushing mechanism predicted by the model. From the viewpoint of organic chemists, the two-stage architecture offers greater transparency compared to single-step approaches, as the arrow-pushing mechanism provides justification for why the final products were predicted.

Siamese Architecture

The two-stage Siamese architecture[16] comprises three distinct models, each serving a specific function. Initially, two separate reactive atom predictor models are instantiated. One model is specifically trained to predict source atoms, while the other is trained to predict sink atoms. To train the source and sink models, the electron-donating atom from the intermolecular arrow is labeled as the source atom, while the electron-accepting atom is labeled as the sink atom. This labeling process employs the reactive sites identification method as detailed in.[16] We convert atoms to fingerprints using predefined atomic and graph-topological features. Subsequently, source and sink classifiers are trained to categorize these feature vectors accordingly. In order to predict reactive atoms, we feed in all the atoms into the source and sink predictors, and rank the atoms by their scores. The top-k source and sink atoms are then thresholded before being paired together to enumerate possible arrow-pushing mechanisms via OrbChain.[13,16,17] Afterward, a Siamese architecture is used as a plausibility ranker model, which then ranks the plausibility of each potential mechanism to generate a final set of predictions. A visual representation of the source and sink pair is provided in Figure 5.

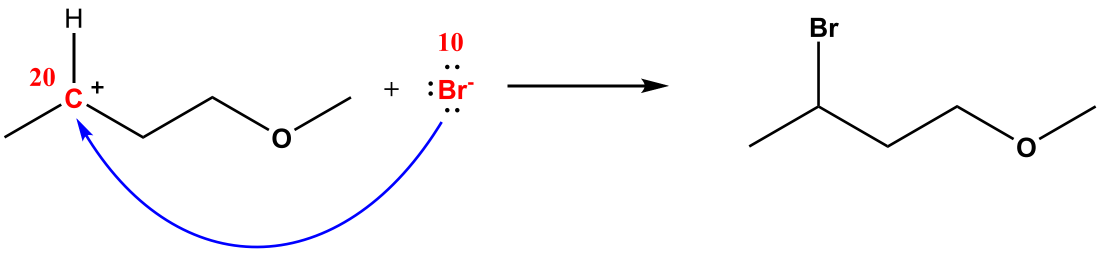

C[CH+:20]CCOC.[Br-:10]>>C[CH:20](CCOC)[Br:10] 10=20

Figure 5: An example of a simple polar elementary step. The electron pushing arrows can be seen in blue, while the source and sink sites are seen in red. The bromine atom labeled 10 is the source atom. The carbon atom labeled 20 is the sink atom. The corresponding SMILES string and arrow codes can be seen below.

Reactive Atom Prediction

On fold0 of the manually curated dataset, a fingerprint of length 6358 is constructed for each atom. This fingerprint consists of 6273 graph-topological features, and 85 hand-crafted physiochemical features. These graph-topological features are extracted using a neighborhood of size 3 with the method described in,[16] while the physiochemical features are derived from properties such as valence number, electronegativity, aromaticity, atomic number, etc. On different folds, the extracted graph topological features are slightly different leading to minor differences in fingerprint lengths.

The source and sink prediction models are trained using the manually curated and mixed datasets. Each training reaction is processed to extract the atom fingerprints. The atom is given a label 1 if it is reactive, and 0 if it is non-reactive. The final output layer performs a binary classification on a reactive atom. The parameters of the source and sink prediction models can be seen in table 8:

Table 8: Source and Sink Model Hyperparameters

Batch Size	Num Layers	Hidden Dim	Dropout	Reg (L2)	Epochs
512	6	540	0.02	1×10^{-3}	100

We assess the performance of the source and sink models on reactive sites identification.

The top-N accuracy of the reactive sites identification on fold0 of the manually curated dataset is presented in Table 9. Reactive site identification is considered correct for an atom prediction model if the source/sink is ranked in the top-k predictions.

Table 9: Reactive Site Identification Accuracies

**Model**	**Top-1**	**Top-3**	**Top-5**	**Top-10**
Source	76.6	94.4	97.5	99.3
Sink	75.7	95.4	97.6	99.1

The source and sink ranking models are able to predict the reactive atoms with high accuracy. Although the reactive atom models are able to filter down the number of potentially reactive atoms significantly, there are some cases where reactions with large aromatic structures generate an extremely large number of reaction mechanisms for the ranker model to evaluate, even if we only generate the mechanisms from 10 source and sink atoms. During model evaluation, we select the top-10 sources and sinks, and then filter away sources and sinks below a threshold in order to reduce the number of mechanisms generated.

Plausibility Ranking

The reactionFP fingerprint is constructed following the methodology of.[16] We begin by extracting the source and sink features, each represented by a vector of length 6,358. Next, we compute the net change features by generating Morgan fingerprints of length 2,048 for both the reactants and the products. The net change fingerprint is then obtained by subtracting the product fingerprint from the reactant fingerprint, resulting in a vector of size 2,048. Finally, we concatenate the net change fingerprint with the source and sink feature vectors to form the complete reaction fingerprint, yielding a final dimensionality of 14,764. To train the ranker, we employ a Siamese architecture: pairs of positive and negative reactionFP fingerprints are passed through a shared feed-forward network, and their outputs are compared to compute a binary cross-entropy loss. Positive examples correspond to plausible mechanisms, while negatives represent implausible ones. At inference time, we no longer use the pairwise

comparison setup; instead, we apply the shared network directly to individual candidate mechanisms, obtaining plausibility scores that allow us to rank mechanisms according to their plausibility.

The parameters of the shared network for the Siamese ranker model can be seen in table 10:

Table 10: Siamese Ranker Model Parameters

**Batch Size**	**Num Layers**	**Layer Dim**	**Act**	**Reg**
2048	3	140-140-140-1	ReLU	Dropout (0.468)

When performing two-step predictions, we select the top-10 source and sink reactive atoms, discard all atoms with scores below a threshold of 0.18, and then generate up to 128 candidate mechanisms using OrbChain. The threshold and maximum number of mechanisms was determined through hyperparameter tuning on the validation set. While using a smaller maximum number of mechanisms can lead to the true mechanism being unrecoverable after source–sink pairing, increasing the maximum number of mechanisms too far caused the ranker to struggle with correctly ranking mechanisms when faced with very large candidate sets.

ArrowFinder

When evalauting the ArrowFinder model, we used fold0 of the mixed dataset. ArrowFinder takes the top-20 source and sink atoms, and enumerates up to 10,000 reaction mechanisms using OrbChain with the reactive atom pairs. In the case of ArrowFinder, we can generate many possible mechanisms because the ranker no longer needs to evaluate a very large candidate set. We automatically filter away the majority of mechanisms because we only save the mechanisms which recover the products. ArrowFinder is able to predict arrows which recover the products in 99.55% of PMechDB test set reactions using this configuration. When reducing the maximum number of reactive atoms to 10, and the maximum number of mechanisms enumerated to 5000, ArrowFinder recovers arrows in 1326 out of 1337 reactions,

leading to a rate of 99.18%. When providing OrbChain with the correct source and sink atoms, it is able to recover the true products 100% of the time. This means the arrow recovery rate can theoretically be increased to 100% on the PMechDB test reactions if the maximum number of mechanisms and reactive atoms is increased indefinitely, but this will always be limited by computing power in practice as it is very expensive to enumerate all possible mechanisms for larger structures. Another less computationally expensive approach would be to improve the accuracy of the source and sink predictors, which directly raises the likelihood that the generated mechanisms will include the ground truth. If we were to always predict the ground truth source and sink atoms, then we could also recover the mechanisms with 100% accuracy. Future work could focus on improving these models to further boost overall recovery.

References

(1) Dashuta, A. E.; Miller, R. J.; Baldi, P.; Sander, T.; Van Vranken, D. L. A Data Set of Plausible Proton Transfer Steps For Arrow-Pushing Mechanisms. *ChemRxiv 10.26434/chemrxiv-2025-64lbf* **2025**,

(2) Crooks, J. E. Proton Transfer to and From Atoms Other Than Carbon. *Comprehensive Chemical Kinetics* **1977**, *8*, 197–250.

(3) Sander, T.; Freyss, J.; von Korff, M.; Rufener, C. DataWarrior: An Open-Source Program For Chemistry Aware Data Visualization And Analysis. *Journal of Chemical Information and Modeling* **2015**, *55*, 460–473.

(4) Guthrie, J. P. Hydration of thioesters. Evaluation of the free-energy changes for the addition of water to some thioesters, rate-equilibrium correlations over very wide ranges in equilibrium constants, and a new mechanistic criterion. *Journal of the American Chemical Society* **1978**, *100*, 5892–5904.

(5) Guthrie, J. P.; Barker, J.; Cullimore, P. A.; Lu, J.; Pik, D. C. The tetrahedral intermediate from the hydration of N-methylformanilide. *Canadian Journal of Chemistry* **1993**, *71*, 2109–2122.

(6) Bernasconi, C. F.; Fairchild, D. E.; Montañez, R. L.; Aleshi, P.; Zheng, H.; Lorance, E. Kinetics of Proton Transfer from Cationic Carbon Acids in Water and Aqueous DMSO. Effect of Activating Groups and Solvent on Intrinsic Rate Constants. *The Journal of Organic Chemistry* **2005**, *70*, 7721–7730.

(7) Shields, B. J.; Stevens, J.; Li, J.; Parasram, M.; Damani, F.; Alvarado, J. I. M.; Janey, J. M.; Adams, R. P.; Doyle, A. G. Bayesian reaction optimization as a tool for chemical synthesis. *Nature* **2021**, *590*, 89–96.

(8) Tavakoli, M.; Miller, R. J.; Angel, M. C.; Pfeiffer, M. A.; Gutman, E. S.; Mood, A. D.; Van Vranken, D.; Baldi, P. Pmechdb: A public database of elementary polar reaction steps. *Journal of Chemical Information and Modeling* **2024**, *64*, 1975–1983.

(9) Joung, J. F.; Fong, M. H.; Casetti, N.; Liles, J. P.; Dassanayake, N. S.; Coley, C. W. Electron flow matching for generative reaction mechanism prediction. *Nature* **2025**, 1–9.

(10) Schwaller, P.; Laino, T.; Gaudin, T.; Bolgar, P.; Hunter, C. A.; Bekas, C.; Lee, A. A. Molecular transformer: a model for uncertainty-calibrated chemical reaction prediction. *ACS Central Science* **2019**, *5*, 1572–1583.

(11) Irwin, R.; Dimitriadis, S.; He, J.; Bjerrum, E. J. Chemformer: a pre-trained transformer for computational chemistry. *Machine Learning: Science and Technology* **2022**, *3*, 015022.

(12) Sterling, T.; Irwin, J. J. ZINC 15–ligand discovery for everyone. *Journal of Chemical Information and Modeling* **2015**, *55*, 2324–2337.

(13) Tavakoli, M.; Baldi, P.; Carlton, A. M.; Chiu, Y. T.; Shmakov, A.; Van Vranken, D. AI for Interpretable Chemistry: Predicting Radical Mechanistic Pathways via Contrastive Learning. *Advances in Neural Information Processing Systems* **2023**, *36*.

(14) Lu, J.; Zhang, Y. Unified deep learning model for multitask reaction predictions with explanation. *Journal of Chemical Information and Modeling* **2022**, *62*, 1376–1387.

(15) Tu, Z.; Coley, C. W. Permutation invariant graph-to-sequence model for template-free retrosynthesis and reaction prediction. *Journal of Chemical Information and Modeling* **2022**, *62*, 3503–3513.

(16) Fooshee, D.; Mood, A.; Gutman, E.; Tavakoli, M.; Urban, G.; Liu, F.; Huynh, N.; Van Vranken, D.; Baldi, P. Deep learning for chemical reaction prediction. *Molecular Systems Design & Engineering* **2018**, *3*, 442–452.

(17) Kayala, M. A.; Azencott, C.-A.; Chen, J. H.; Baldi, P. Learning to predict chemical reactions. *Journal of Chemical Information and Modeling* **2011**, *51*, 2209–2222.
